# Scientific mistakes from the agri-food biotech critics

**DOI:** 10.1186/s40504-018-0089-7

**Published:** 2018-12-10

**Authors:** Giovanni Tagliabue

**Affiliations:** Carugo, Como Italy

## Abstract

Critics of the use of advanced biotechnologies in the agri-food sector (“New Breeding Techniques”, comprising CRISPR) demand a strict regulation of any such method, even more severe than rules applied to so-called “Genetically Modified Organisms” (i.e. recombinant DNA processes and products). But their position is unwarranted, since it relies on faulty arguments.

While most life scientists have always explained that the trigger for regulation should be the single *product* and its phenotypic traits, opponents insist that the target should be certain biotech *processes*.

The antagonists maintain that NBTs are inherently risky: this belief is exactly the opposite of a long-standing, overwhelming scientific consensus. NBTs involve unpredictable effects, but it is the same for the results of any other technique. The critics wrongly equate “unintended” with “harmful” and misunderstand two meanings of “risk”: the “risk” of not achieving satisfactory results does not automatically translate into health or environment “risks”. Generic claims that allergenic or toxic properties are a hidden danger of outcomes from NBTs are unsubstantiated – as they would be for traditional techniques.

Among several errors, we criticize the misuse of the Precautionary principle, a misplaced alarm about “uncontrolled spreading” of genetically engineered cultivars and the groundless comparison of (hypothetical) agricultural products from NBTs with known toxic substances.

In order to “save” traditional techniques from “GMO”-like regulations, while calling for the enforcement of similar sectarian rules for the NBTs, the dissenters engage in baseless, unscientific distinctions.

Important and necessary socio-economic, ethical and legal considerations related to the use of agri-food biotechnologies (older and newer) are outside the scope of this paper, which mostly deals with arguments from genetics, biology, and evolutionary theory that are provided by those who are suspicious of NBTs. Yet, we will provide some hints on two additional facets of the debate: the possible motivations for certain groups to embrace views which are utterly anti-scientific, and the shaky regulatory destiny of NBTs in the European Union.

## Introduction

In this paper we aim to debunk some basic conceptual mistakes regarding criticism of the use of genome ameliorations in the “green” (i.e. agri-food) biotech area, in particular with regard to the very recent, in-progress group of methods generically indicated by the term “New Breeding Techniques” (“NBTs”), comprising CRISPR. Other biotech areas, e.g. “red” (i.e. medical-pharmaceutical), in particular the use of gene editing to delete or correct detrimental mutations in the human DNA, or the implementation of “gene drives” to reduce populations of noxious animals (e.g. mosquitoes or rodents) or plants (e.g. invasive weeds) in open environments, raise different/additional problems, both theoretical and ethical. We circumscribe our focus to issues related to agronomy, discussing some of the underlying genetics, biology and evolutionary theory concepts.

In order to show that skeptics of advanced agricultural biotech methods try to base their call for strict regulation on views which are unscientific, misunderstood or even plainly contradictory, we examine three documents (ENSSER 2017; Steinbrecher and Paul 2017; Then and Bauer-Panskus, 2017) recently issued by organizations and authors which have a historical record of opposition to recombinant DNA (rDNA) techniques, i.e. the “classic” transgenesis, used to produce so-called “genetically modified organisms” (“GMOs”).^1^ We dissect the wrong assertions that are supposed to be a sound scientific basis for the agri-food regulation of NBTs. The same fallacious arguments which have been put forward for decades against rDNA operations and products are now explicitly extended to the newest techniques. Being devoid of epistemological value and bereft of empirical evidence, the demand to enforce severe restrictions on NBTs *as a whole* has no grounds.

Therefore, this paper is *not* about outlining a regulation of NBTs or other groups of techniques (rDNA, mutagenesis, etc.), but rather about the life-sciences background that must be correctly set out in order to debate what regulatory approach is more adequate – which is a following step, beyond the remit of this text. In other words, we are not calling for certain *ethical or normative attitudes* (e.g. a cost-benefit approach) to be – or not to be – a beacon in the agri-food biotech regulation; our aim is to point out that any such (necessary) debate is warped, if basic scientific concepts are got wrong.

Our criticism is logically placed *before* any risk management perspective: if some keystones of genetics/biology and the same notion of risk are mistaken, we cannot even start discussing actual risks and how to deal with them. In other words, we are not debating values that can be related to the use/abuse/misuse of NBTs: we try to point out that anti-biotech critics are wrong in understanding what we are talking about in the first place. Therefore, the bone of contention in this paper is not whether NBTs are good or bad, risky or safe (a question that cannot be applied to NBTs as a group of diverse techniques, loosely labelled under the same acronym), but what life scientists explain them to be – and what biotech detractors fail to grasp.

These opponents place an exclusive, almost obsessive emphasis on (misunderstood) risks and dangers: such a totally negative attitude reveals a biased, unescapable anti-biotechnological mindset, which seems to influence the theoretical misapprehension of the matter. We hope that our constructive criticism may encourage a rebalanced disposition.

## Background: An ongoing revolution in genomics, biology and agronomy

In recent years, various advanced methods of intervention for modifying the genomes, and consequently improving the phenotypic characteristics, of living objects (plants, animals, microorganisms, fungi) have been developed, with important applications also in the “green” sector: collectively, they are named “New Breeding Techniques”, “NBTs”^2^ (European Commission, Scientific Advice Mechanism 2017, p. 56–75. For a less technical explanation, see EASAC 2015). The not yet well-assessed perimeter of NBTs may include synthetic biology, i.e. the creation of genes which do not exist in nature. A brief explanation of the many properties and features of NBTs is unfeasible, since esoteric technicalities should be clarified; but such an in-depth analysis is unnecessary to our discussion.^3^ Suffice it to say that these methods, above all CRISPR, allow rapid, less expensive and more precise modifications and enhancements of agri-food novelties: hence the widespread excitement among life scientists and breeders all over the world.

Some of these techniques, also according to details in their different applications, may be indicated as “gene editing” or, better, “genome editing”: this expression usefully suggests an analogy between the cut-and-paste, or even deletion, of DNA sequences,^4^ with similar operations that can be carried out on written phrases using a word processor; such a comparison extends the recurrent metaphor of the genome as a text, whose letters are the four bases (A, T, C, G).

In certain cases, these interventions create transgenic organisms, i.e. some sequences of exogenous DNA are permanently infused in the genome of the new organisms (only the methods – and their precision - differ from those used in former transgenic achievements); in other situations, the insertion is only provisional, in that it is useful during the lab work - the resulting phenotype is not transgenic; or there is not an insertion but a deletion of DNA portions; or the genome is not changed, but epigenetically influenced, so to say, in order to “switch on or off” certain genes; in these last three cases, the new organisms are often indistinguishable from those obtained via traditional techniques (e.g. crossing, physical/chemical mutagenesis) or natural mutation. (John Innes Centre 2015).

As of today, myriad proofs of concept regarding new organisms created through NBTs have been described for model plants, crop plants, fruit plants, woody plants, vegetables and grasses, e.g. alfalfa, barley, potatoes, poplar trees, petunias, rice, lettuce, soybeans, sorghum, tomatoes and lemon trees (Tang, Tang 2017). In addition: wheat that is resistant to powdery mildew (a destructive fungal disease); wheat made edible for celiacs (Saplakoglu 2017); corn and wheat strains edited for drought resistance. As for animals, an already outdated list indicates “more than 300 differently edited pigs, cattle, sheep and goats” (Tan et al. 2016). A few products generated via NBTs have recently been authorized in the USA: cultivars of potato, apple and mushroom which resist browning will soon be marketed. The first pasta with genome-edited cabbage has been cooked and tasted (Cohen 2016). Many hundreds of beneficial products are in the R&D pipelines of universities and companies, and they can become thousands in a matter of years - not decades, as for conventionally bred plants and animals.

## Distinguishing biotech areas

A first explanatory step must make clear a necessary distinction between different areas/“colors” of biotechnologies: “red” (medical-pharmaceutical), “green” (agri-food), “white” for industrial applications, “grey” for bioremediation and “black” for biological warfare and bioterrorism.^5^ The partition of the different sectors of biotechnologies is indispensable: in the “green” area, an approach which focuses on biotech process(es) as the trigger for regulation is illogic and deleterious, while in other areas it may not be so. Instead, opponents of NBTs fail to make that necessary separation: their demand is to “bring the regulation of NGMT applications in agricultural and other contexts into line with their recognition in the sphere of medical research” (ENSSER 2017, p. 10): but crops or foods/feeds/fibers and drugs are radically different: dealing with pathogens (e.g. infective viruses or lethal bacteria) in the “red” or “black” biotech sectors requires rigid safety procedures; similarly, the release of, say, oil-degrading bacteria in an open sea (an example of “grey” biotech operations) must entail great circumspection. Instead, images of biotech foes who invade fields of “GMO” maize wearing hazmat suits are only good for media stunts – although we must recognize that the antis’ antics are quite impressive for those who are not aware of the merely propagandistic motivations of those acts.

Ironically, a real danger is inherent in the use of a double agricultural biotechnology which nobody is worried about: in experiments of physical/chemical mutagenesis, operators must be very careful while using radiation and nasty chemical substances – a peril that does not exist with rDNA or NBT operations, neither in labs nor in greenhouses or open fields.

## Product, not process: a basic concept

The alleged scientific reasons which should support the demand for strict regulation of agri-food NBTs boil down to a few mistakes or misunderstandings: the first one is the somewhat worn-out issue which is called “product vs. process” debate. It is the discussion on whether regulators, when considering green biotechnologies, should focus on the actual characteristics of the phenotypic outcomes, i.e. the plant or animal or microorganism that has been endowed with new trait(s) – the *product*, the real thing – and/or on the *process(es)*, i.e. the methods which have been used by experimenters to produce this or that agricultural novelty.

The origin of this question dates back to the early 1980s, when progress in molecular biology showed the potential of using recombinant DNA techniques to change some small areas in the genomes of various cultivars (the applications directed at modifying animals came later), often adding nucleotide sequences which were called transgenes, in that they were “copied” from other organisms (more often other plants, sometimes bacteria or viruses). The aim is to obtain various results: fight diseases, repel pests, provide herbicide tolerance, enhance nutritious properties, extend shelf life. Similar, even identical phenotypic effects had been obtained with existing techniques (mostly crossing and physical/chemical mutagenesis), but the new applications may allow the species barrier to be overcome. This major biotech advance was seen by breeders as a great opportunity: direct intervention on the genomes, although with methods that are imprecise (bacterial or viral vectors, “gene guns”), opened up a whole new scenario for the improvement of plants, for both food and non-food uses.

The same perspective that was considered so positive by most life scientists and agronomists was soon exploited by anti-biotech groups to start their relentless, very successful opposition: the expression “genetically modified organisms”, which is scientifically meaningless, was coined as a very pejorative term, and the technology (the *process*) was charged with vociferous accusations of hidden dangers to health and the environment. No attention was paid by opponents to the actual characteristics of genetically improved outcomes (the *product*), no subtleties were allowed: “In agricultural crops, products of rDNA technology were lumped together into one ominous category, regardless of trait, genetic event, or species.” (Herring 2010, 80) Yet, as we will see, according to the widespread scientific consensus,^6^ there are no theoretical and empirical justifications to view as separate domains the older, and still valid, breeding techniques (including mutagenesis) from “GMOs” and NBTs – some of which, however, are mutagenetic methods.

Since the very beginning of the process vs. product debate, the view of most life scientists and breeders has been unequivocal: the focus on the process(es) as a trigger for regulation, as regards both safety and environmental impacts, is not valid. The epistemological basis of this uncompromising position is neat: what counts is “the nature of the organism and the environment into which it will be introduced, not the method by which it was modified.” (National Academy of Sciences 1987, p. 7) “Genetically engineered organisms should be evaluated and regulated according to their biological properties (phenotypes), rather than the genetic techniques used to produce them.” (Tiedje et al. 1989, p. 298). Hence the following valid step, which broadens the same view to the NBTs: “it is not possible to provide a scientifically sound safety assessment of breeding techniques as such. The safety assessment can evaluate the properties of each specific end-product only on a case-by-case basis.” (European Commission 2017, p. 77).

Scientists were hopeful that lawmakers would take into account their clear-cut position. Of the many explicit calls in that sense, consider the plea from the European Molecular Biology Organization, which dates back to 1988: “EMBO strongly believes that there is no scientific justification for additional, special legislation regulating recombinant DNA research *per se*. Any rules or legislation should only apply to the safety of products according to their properties, rather than according to the methods used to generate them.” (40th meeting of the Council of the EMBO, cit. in Morris and Spillane, 2010, p. 361, n. 16). Also at international level, a basic document issued by the OECD was very explicit: “There is no scientific basis for specific legislation for the implementation of rDNA techniques and applications.” (OECD 1986, p. 42).

These appeals went unheeded: in many countries, and internationally, politicians created a “GMO” regulatory ghetto which is still in place. By the beginning of the new millennium, laws were established with the declared purpose of severely regulating – to the point of heavily restricting, or even banning outright – the *process* of genetic engineering in the agri-food area. Also at the international level, most countries adhered to the Cartagena Protocol on Biosafety, with its basic suspicion regarding the alleged possible dangers of just *transporting* rDNA commodities. A sound product-based legal framework for agri-food biotechnology was only implemented in Canada and – initially and still partially – in the USA.

To be clear, if other groups of products, e.g. those 3000+ varieties obtained via mutagenesis (FAO-IAEA 2018) were singled out to be governed apart for the sole reason that they derive from certain processes, the rationale would be equally anti-scientific: yet, only “GMOs”, in most regulators’ eyes, form a caste that merits unwarranted suspicion.

Note that the adoption of a product-based criterion does *not* mean that the regulation should generally be looser: decision-makers may decide to subject breeders, both in private companies and in public institutions, to more lenient or stricter rules (e.g. fewer or more analytical pre-market tests, in the labs and/or in the fields or farms): but the established guidelines should assess the different levels of known or rationally foreseeable risks (allergenicity, toxicity, invasiveness), depending on the traits and characteristics of the product. Today, instead, any rDNA cultivar is, in almost all jurisdictions, trapped inside a regulatory nightmare which exempts very similar “non-GMO” varieties (e.g. mutagenized or hybridized herbicide tolerant crops).^7^ “In all of these scenarios involving process-based triggers, limited regulatory resources are expended without any consideration of actual risk and without increasing the actual safety to the public or the environment.” (McHughen 2016, p. 131).

Yet, according to those few who believe that it is necessary to adopt the process-based perspective, supporters of NBTs are reportedly willing “to grant them ‘light-touch, product-based’ regulated status” (ENSSER 2017, p. 2). This remark is baseless: by just looking at the framework which is outlined by the many proponents of product-based regulation (see e.g. McHughen 2016, Tagliabue 2017), it is obvious that equating “product-based” regulation to “light-touch” regulation is far from reality. Moreover, a much worse misunderstanding is affirming that product-based regulation “focuses only on the intended outcome of a *theoretical* intervention *into the genome*.” (ENSSER 2017, p. 2, our emphases) This is simply contradictory: indicating products and their actual characteristics as the appropriate target for regulation means that the outcomes to be assessed are not “theoretical” but real, existing, examinable; and the objects of the necessary evaluations are not primarily the genomes (although the study of changes at molecular level is certainly instructive), but the phenotypes, i.e. the products. Here, the product-process issue is completely misinterpreted by NBT critics.

An alleged defect in the product-based regulation prospect is pointed out by skeptics: “Only the intended trait present in the end product of the NGMT ‘event(s)’ should be considered by regulators, and no attention should be given to the processes by which these ‘events’ were created within the entire organism, whether a virus, microbe, plant or animal.” (ENSSER 2017, p. 2–3) The attribution to supporters of the product-based approach of a narrow focus on the new trait, and not on whether it is well integrated in the target organism, is dubious: this alleged lack of a “holistic” perspective seems to hint at a possible regulatory weakness. We believe that the necessary tests and exams which are mandatory in the product-oriented framework should appease that concern: it can be quieted by writing a regulation that imposes assessment of the relationship between the new trait(s) and the whole phenotype – a notion that is logically implied in a science-informed viewpoint. Sets of reliable tests, such as those provided and governed by the Codex Alimentarius (an institution linked to the World Health Organization and the Food and Agriculture Organization) and similar science-based authorities (e.g. the European Food Safety Authority) are in place. It is interesting to note that the Codex’s procedural manual for comprehensively assessing the safety of food is constantly updated, even when small additions or changes seem necessary (Codex Alimentarius 2016), while the guidelines for “Foods derived from modern biotechnology”, i.e. “GMOs” (plants, microorganisms and animals) were established once and for all (Codex Alimentarius Commission 2009): it makes sense, because a careful reading of those indications shows that they are by no means different from those recommended for food in general.

Another contorted sentence must be broken down: “the claim that the new techniques are more precise therefore more controlled, and that this justifies no regulation of the process, only of the final product, neglects all the scientific evidence” (ENSSER 2017, p. 8). We believe that this is a wrong explanation: the call to focus only on the product, and *not* on the process(es), as the logical, science-based trigger for regulating the agri-food biotechnologies, is completely independent from the more or less precise character of the different applications. In other words, in the product-based approach, *any* organism that derives from *any* technique (from hybridization – comprising “wide crosses” – to grafting, cell fusion, polyploidy induction, physical/chemical mutagenesis etc.) should be evaluated on its own pros and cons: NBTs are actually more precise, but the safety and environmental impact of their outcomes (the products) must be assessed individually. Thus, the plea to consider only the product is not a call for general de-regulation, but for a shift from an incoherent perspective to a rational one.

## A basic misunderstanding about the concept of “risk”

Since the appeal to regulate the process – rather: only certain arbitrarily circumscribed techniques – is invalid, one may wonder where the strenuous call to regulate NBTs as “GMOs” has its real basis: the answer is surprising, because the alleged motivation lies in the “risks inherent in the genetic modification process” (ENSSER 2017, p. 2).

This is certainly the most unscientific stance offered by the radically anti-biotech fringe: instead, the mainstream position among life scientists affirms that “conjectural risks of genetic engineering must be of the same order as those for natural biological evolution and for conventional breeding methods. [...] There is no scientific reason to assume special long-term risks for GM crops.” (Arber 2010, Abstract. For a reference list of many similar positions, see Tagliabue 2016) This is not only an epistemological keystone. Hundreds of scientists were tasked by the EU to make a widespread, in-depth analysis of the scientific status of rDNA agri-food techniques; after a number of research projects during the period 1985 to 2000, many others were carried out in the decade 2001–2010: “The main conclusion to be drawn from the efforts of more than 130 research projects, covering a period of more than 25 years of research, and involving more than 500 independent research groups, is that biotechnology, and in particular GMOs, are not *per se* more risky than e.g. conventional plant breeding technologies.” (European Commission 2010, p. 16) Clear-cut position statements affirming the same principle were issued by several scientific societies all around the world; see e.g. a petition signed by 3400 scientists – inter alia 25 Nobel laureates: “The risks posed by foods are a function of the biological characteristics of those foods and the specific genes that have been used, not of the processes employed in their development.” (Prakash et al. 2000–2014).

Thus, the main argument to justify the demand to treat NBTs like “GMOs” clashes with a central tenet of contemporary genetics and biology and claims to dismiss mountains of evidence. As the saying goes, extraordinary claims need to be supported by extraordinary discoveries; yet, the truth is probably much more trivial, if we consider the following para-statistical piece of information regarding the outcomes from NBTs: “the success rate fluctuates strongly according to method, type of cell and organism - an unmistakeable sign that the methods are still associated with many *risks and uncertainties*.” (Then, Bauer-Panskus 2017, p. 4, our emphasis) This statement shows that, when the antagonists talk about “risk”, they confuse the high probability of failure of the attempts by breeders to obtain the hoped-for results – i.e. a new organism endowed with the desired trait – with the “risk” that such frequent unsatisfactory outcomes always represent a health or environmental danger.

The impression regarding such confusion becomes a certainty if we consider that the demand to avoid any safety “risk” becomes, for the hypercritics of NBTs, an unreasonable quest for full predictability of the experiments: “the question remains about whether all the risks will be discovered before organisms are released.” (Then and Bauer-Panskus 2017, p. 17). In other words, “we do not yet know all the mechanisms by which these methods bring about changes in the sequence of DNA, nor to what extent these may differ between animals and plants, or subgroups. This undermines our ability to fully predict the outcomes of these procedures.” (ENSSER 2017, p. 4) We must repeat a simple truth: the changes in the genome that “we don’t know” and the impossibility of “fully predicting” the outcomes of the experimental attempts, apply to *any* agricultural biotechnology, not only to NBTs (and “GMOs”); but this partial ignorance is not a synonym of inability in assessing agri-food novelties. If anything, the level of unpredictability is higher with traditional methods, but this won’t discourage breeders from trying them, insofar as they appear to be promising in this or that situation. Thus, we are not downsizing the valuable contribution that can be made to agriculture by older, still widely used techniques: sometimes, non-transgenic outcomes perform better (Gilbert 2014); when traditional solutions are available and work more effectively, all else being equal, farmers may quickly adopt them, even abandoning similar transgenic products - and unbiased observers would acknowledge the progress.

## The false problem of “unintended changes” in the genomes

We do not maintain, as some over-confident life scientists occasionally claim, that “the rDNA technology is safe”: no technology, in any field, can be declared to be inherently devoid of risks. Yet, we do not need preliminary, impossible certainty about the infallibility of this or that “green” biotech method: because, when a tryout – *“GMO” or otherwise* – proves to be unsatisfactory, it is abandoned; that is exactly what experimenters have done in various cases, discarding ill-fated rDNA varieties (e.g. soybeans, barley, canola, maize, potato, rice, wheat, flax, corn, etc.) *and* traditional ones (e.g. squash, celery, and potato) (Haslberger 2003, p. 740; Kuiper et al. 2001, p. 516; Colorado State University 2004). For breeders - now as in the past, and all the more so in the future - the results of any attempt to create new races or varieties must be assessed a posteriori, through valid tests which are available. There is no reason why this dynamic should not be valid for the products obtained from any breeding technique, present and future.

This clear, evidence-based position is also intended to defuse the recurrent claim of the antis, when they maintain that the unpredictability of the results from NBTs and the frequent off-target effects automatically translate into safety/environmental risks. On this subject, a third text that argues for the strict regulation of agri-food NBTs is instructive about the link between semi-truthful descriptions, tendentious wording, incoherent reasoning, bad examples and wrong conclusions.

We are reminded how, during rDNA operations, many parts of the genome in the target organism are involved in changes that are not related to the correct integration of the transgenes into the expected loci, and warned that “mutations as small as one DNA letter (a point mutation) can result in missing or malformed proteins with potentially severe consequences.”^8^ (Steinbrecher and Paul 2017, p. 39–40) The authors’ conclusion is that “[i]n the production of GMOs, there are many stages in the whole process where something unforeseen may occur.” (Steinbrecher and Paul 2017, p. 39) The usual amendment is needed to transform a half-truth into a fully correct statement: just replace “GMOs” with “any agri-food novelty - with any technique”. If this clarification is not made, when it is pointed out that “such mutations also occur with the new genetic engineering techniques” (Steinbrecher and Paul 2017, p. 39), the reader who does not know better will be under the disturbing impression that these hidden genomic movements and changes are disrupting processes (they are) which must be *necessarily* “risky”, i.e. harmful (they are not): this inconsequential remark should not create any generic, pre-emptive anxiety about breeding activities, older and newer. So, “the recognition that the process of genetic engineering itself can give rise to unintended changes at the DNA level”, contrary to the intention of the authors, will not induce us to ponder only on its “potential negative effects” (Steinbrecher and Paul 2017, p. 40).

Thus, the misunderstood concept of risk and the inadmissible request for utmost predictability are linked to another source of skepticism, a semi-truth from which opponents of NBTs erroneously deduct negative consequences: “unintended changes in the genome occur frequently when *these* techniques are applied to some organisms and have not been excluded as happening in any organism” (ENSSER 2017, p. 4, our emphasis). The usual operation to amend the recurring bias is needed: replace “these” with “any” and the statement will be correct. In other words: “no system for genetic modification*, including conventional methods of plant breeding*, is without unintended effects. [...] ‘unintended’ does not necessarily mean ‘harmful’.” (Ladics et al. 2015, Abstract, our emphasis). Indeed, the basic definition of “genetic modification” is “the alteration of the genotype of a plant using any technique, new or traditional”. (FDA 1992) The same for microbes: a widely used handbook makes no distinction among organisms that are “wild type” or that have been genetically modified by one or another method. (National Institutes of Health 2009).

Consequently, the mantra which demands severe restrictions, repeated for the nth time, is unsupported: the conclusion “All products of NGMTs must *therefore* be regulated at the level of strictest GMO regulations” (ENSSER 2017, p. 7, our emphasis) contains a “therefore” that has no justification.

The only specification of the alleged dangerous nature of undesired changes is shaky. It starts with a repeated misunderstanding: “proteins that are changed in their structure may form. This can lead to the emergence of unexpected and surprising biological effects in the cells or the organism that are not immediately *predictable at the DNA level*.” (Then and Bauer-Panskus 2017, p. 6, our emphasis). The prediction of changes inside the genome is the least of breeders’ concerns: as we have already pointed out, the analysis of the genomes resulting from experiments is scientifically interesting, but those who strive to create enhanced agri-food novelties are mostly – and very reasonably - interested in the actual phenotypic characteristics of the outcomes. Furthermore, “plant mutagenesis may induce more transcriptomic changes than transgene insertion.” (Batista et al. 2008; see also Vincelli 2018, offering several references) “The ‘-omics’ comparisons revealed that the genetic modification has less impact on plant gene expression and composition than that of conventional plant breeding. Moreover, environmental factors (such as field location, sampling time, or agricultural practices) have a greater impact than transgenesis. None of these ‘-omics’ profiling studies has raised new safety concerns about GE varieties; neither did the long-term and multigenerational studies on animals.” (Ricroch 2013, Abstract) “[W]hen the safety implications of genetic disturbances such as insertional mutagenesis, deletions, duplications, and the creation of potential chimeric genes are considered for GM crops, these assessments should consider that such genome disruptions occur naturally and frequently during plant domestication and traditional breeding, and do so without any recognized safety implications.” (Parrot et al. 2010, p. 1777) Thus, again, the expected genomic changes become a matter of concern only if, and to the extent that, the safety and environmental assessments of a particular new organism – from *any* technique – appear problematic: and this, needless to repeat, depends on phenotypic characteristics of the organism, not on the process used in its creation.

When an effort is made to clarify the possible danger from the NBTs, recurrent dubious assertions are offered: “there can be an increase in allergenic plant constituents.” (Then and Bauer-Panskus 2017, p. 6); “off-target effects can lead to unexpected toxins or allergens, or altered or compromised nutritional value” (ENSSER 2017, p. 5). Generic, sweeping statements like these are necessarily unsubstantiated (Bonham 2013): no reference is given, for the simple reason that the structure of toxic proteins is well studied, and therefore the sudden appearance of noxious properties in new cultivars is very improbable: it is “highly unlikely that modification of amino acid sequences can make a non-toxic protein toxic.” (Hammond et al. 2013) Bad surprises are hardly possible, both in rDNA (“enhanced genetic instability from a transgene or from common sequences in two or more transgenes is remote”) and in mutagenesis (“There is no evidence that a random genomic change in a crop has resulted in a novel food or feed safety issue”) (Steiner et al. 2013, p. 1587; see also Weber et al. 2012). The epistemological rationale behind this evidence is well-established: “the potential adverse health effects arising from biotechnology-derived foods are not different in nature from those created by conventional breeding practices for plant, animal, or microbial enhancement, and are already familiar to toxicologists. It is therefore important to recognize that the food product itself, rather than the process through which it is made, should be the focus of attention in assessing safety.” (Society of Toxicology 2003, p. 2). Sounds like a concept we have already come across…

Therefore, the biased admonishment that we can summarize as “beware new allergens – but only from GMOs and NBTs”, repeated ad infinitum by the antis, has no basis in the existing scientific consilience. Specific guidelines to deal with allergens in biotech-enhanced foods are available (FAO-WHO 2001; EFSA 2017): not surprisingly, these instructions do not differ from the ones which are prescribed for *any* novel food.

Remarkably, two rDNA experiments resulted in allergenic outcomes: a soy variety was created with the insertion of a gene from a Brazil nut, in an attempt to enhance the content of methionine (an essential amino acid) to improve feed, but the preliminary tests showed possible problems (Nordlee et al. 1996); a gene was copied from a bean to a pea variety to provide resistance against a major insect pest, but the effectiveness of the pesticide action was accompanied by some allergenic effects (Prescott et al. 2005).^9^ Of course, these failed products never reached the market. The antis accurately avoid quoting these cases,^10^ presumably because they prefer not to point out that the available tests do their job in identifying real problems.

On the contrary, genetic engineering interventions can reduce or eliminate the well-known allergenic potential of peanuts (Rowe 2008), soybeans (Herman et al. 2003) and other vegetables: “GM offers the potential of removing the allergenic or toxic elements from some food crops, thereby preventing many needless deaths, for example removing the allergenic traits of the peanut and the cyanide properties of cassava” (IUCN 2007, p. 32). Those who are so worried about the alleged possibility that “GMOs” can cause allergies should support such efforts. But they do not. They just aim to extend the scope of a prejudice, from “GMOs” to NBTs, without distinctions.

Let’s look at another “unintended effect” which seems to become a serious issue, i.e. “that the DNA for the gene-scissors is inserted into the genome of the plant cells is recognised by experts as a general problem.” (Then and Bauer-Panskus 2017, p. 15) Statements like this one abound in the anti-biotech publications: the reader is induced to think that the experimenters who are pointing out the difficulty are worried about harmful consequences. Not so. They are simply indicating a *technical* issue, i.e. the advantage of eliminating the remains of exogenous DNA from the final product, after its insertion during the transformation process: even if inactive, those leftovers are disturbing in the regulators’ mindset. Yet, if no unfounded taboo with the residues of harmless provisional DNA existed, this “problem” wouldn’t even have been invented. In other words, many scientists are trying to escape the legalistic, scientifically nonsensical “GMO” iron cage, by using CRISPR or similar techniques, without leaving remains: that would be a nice “*delitto perfetto*”, i.e. a crime that leaves no trace (Storici and Resnick 2006). Thus, the NBT critics transform scientists’ remarks on operational issues into gratuitous sources of uneasiness.

## Genomic changes, herbicide tolerance and environmental pollution

The unfailing and fallacious indication of a supposed link between “genomic changes” and “risk” (i.e. danger) is allegedly supported by only one example of bad effects of rDNA, which is introduced by a sweeping declaration: “As first-generation GMOs already showed, the trait of herbicide tolerance can have profound impacts on the environment, human health, biodiversity, and socioeconomic conditions.” (Steinbrecher and Paul 2017, p. 43–44) Here we have a number of mistakes in one statement: a. Pointing to “herbicide tolerance” (HT), i.e. to the use of crops that can survive the spray of weedkillers (therefore facilitating the work of farmers) as exclusive to “GMO” is wrong: there are plenty of HT cultivars which are not rDNA (Reddy and Nandula 2012); b. Furthermore, the diffusion of crops endowed with this useful trait dates back several decades, long before any HT “GMO” crop had been invented; c. HT is one trait among many, and very different: there is no justification in considering it (singular) as representative of “GMOs” (plural): here, the illogical jump from the alleged noxiousness of a single trait to all *products* that are created through certain *processes* is evident. Several HT crops derive from mutagenesis, a few HT crops were obtained by crossing, and some HT crops are naturally tolerant to certain weed killers: yet, we do not see them as proofs of the inescapable dangerousness of traditional biotech methods, as well as of Mother Nature.

But it gets worse: the only supposed demonstration of the alleged gloomy “profound impacts” of “GMO” at various levels, which are declared as evidence, is in a study that, in the words of its authors, reports “high glyphosate pollution in association with increased frequencies of cancer in a typical argentine agricultural village, *and by design, cannot make claims of causality*.” (Avila-Vazquez et al. 2017, Abstract, our emphasis) So, reference is made to a paper that explicitly declares its inability to establish a causal link between a weed-killer and cancer, also because the reported environmental pollution was likewise due to “other pesticides” (Avila-Vazquez et al. 2017, Conclusion); furthermore, the journal’s publisher has a dubious reputation (it is listed at http://beallslist.weebly.com/. Accessed 20 November 2018). Instead of relying on a single clumsy source, Steinbrecher and Paul could have listed several documented cases of “GMOs” which fell short: unbiased, pro-science scholars have no problem in recognizing that also rDNA breeding experiments most often fail, that unsatisfactory results are part of the game in the green biotech world.

Thus, we understand that the real target of this example is glyphosate – the most hated target of the antis today. Note that: a. The product has been marketed since 1974, when HT rDNA crops were still more than twenty years away; b. Its adoption is not necessarily linked to HT rDNA varieties: in the EU, where the cultivation of “GMOs” is practically forbidden, this weed-killer – now off-patent – is largely used. In the space of a few paragraphs, we have witnessed an attempt to establish a link between a declaredly harmful pseudo-category (“GMOs”) and one trait (HT) that is not limited to rDNA, illustrated by an irrelevant paper whose shortcomings are not indicated, with the intent to attack a herbicide that has been exploited for decades before the advent of rDNA crops and that in vast agricultural areas of the world has nothing to do with “GMO”. And it is impossible to understand how the sidelong denigration of one agricultural tool can be logically linked to the generic suspicion regarding NBTs – a various group of techniques that can produce agri-food products as diverse as can be imagined.

Beyond the poverty of this convoluted argumentation, we had better make clear that environmental problems are real: if and to the extent that an agricultural practice proves to be noxious, it must be corrected, even prohibited – *“GMO” or otherwise*. Yet, this necessary action will not cast a general, unsubstantiated doubt on other products or courses of action which are very different and possibly – or certainly – beneficial: virus-immunized papaya or nutritionally-enhanced cassava or submersion-tolerant rice – all “GMO” – have nothing to do with environmental pollution; one of the most widespread rDNA traits (insect resistance) has been very advantageous to the environment and farmers, lowering the use of pesticides and requiring less work in the fields (Brookes and Barfoot 2017); and several NBT applications may contribute to managing problematic agricultural-environmental issues.

## The myth of “uncontrolled spreading”

Another “risk” which is strongly underlined is supposedly due to the fact that “GMOs” “are living systems with the ability to self-replicate and spread their genes *far and wide* through pollen and seed” (Steinbrecher and Paul, p. 40, our emphasis). Yet, in “green” agricultural operations, none of the traits which can be deleted (e.g. elements of toxicity or allergenicity) or added (e.g. resistance to drought or diseases, improved nutritional content, herbicide tolerance, pest resistance) – with *any* biotechnique - can fuel uncontrolled spread.

To understand this, we have to focus on a general characteristic of domesticated species: cultivars or farmed animal strains are almost always *weak*, i.e. they have a hard time in surviving without human support. This ineluctable reality is linked to the fact that any such variety is a product of artificial selection, i.e. it has been singled out and taken care of, most often lowering its natural defences. Many plants have had their poisonous properties bred out through centuries of cultivation, but “reducing the content of natural toxins is a trade-off process: the lesser the content of natural toxins, the higher the susceptibility of a plant to pests and therefore the stronger the need to protect plants.” (Morandini 2010, p. 482) Leave a cultivated field or a vegetable garden neglected, even for a short period, and it will inevitably be overgrown by weeds, which are tempered by natural selection (in passing, we may note that this is precisely the reason why herbicides are an indispensable tool for farmers, i.e. they can avoid hand-weeding and/or deep ploughing to get rid of stubborn weeds): “crop plants typically do not have the characteristics of invasive species, being highly dependent on humans for their survival”. (IUCN 2007, p. 27). That is why in the long list of invasive species there is not a single cultivar, let alone “GMO”. (http://en.wikipedia.org/wiki/List_of_globally_invasive_species).

As for animals, domesticated species were chosen also for their limited aggressiveness, and their docility was amplified during millennia of farming: cattle or poultry that escape farms will be easy victims of predators. Moreover, invasive species of animal or plants have almost always been *natural* species (e.g. rodents or weeds), unwillingly or willingly introduced in different environments. Transgenes may occasionally pass from some rDNA plants into relatives where they were not intended to be infused: in such cases, if and to the extent that the plant becomes invasive, the phenomenon must be contained – even with measures designed to eradicate it. Indeed, this is a problem which is not related to the origin of the cultivar which has acquired weediness, i.e. it does not depend on the process through which it was obtained.

To explain this basic point, consider canola, a modified variety of oilseed rape that was invented at the beginning of the 70s, by Canadian breeders, through selective crosses; in the 1990s and until now, several rDNA varieties of canola were created with added traits, e.g. herbicide tolerance. In North Dakota, the presence of the plant in several places outside fields was noted: which is no surprise, given the weedy characteristic of the original species and its many derivatives, that can thrive also in the wild. It was ascertained that many of those spontaneous plants were “GMO” (Biello 2010): does it matter? In those vast agricultural areas, cultivation of *both* “conventional” and rDNA canola are widespread: if the quantity of the volunteer bushes becomes a problem, actions would be put in place – *whether the weeds are rDNA or not*.

It is the same for occasional hybridizations of plants of different varieties in adjacent fields, even if some of these spontaneously crossed plants are rDNA. The “gene escape” – another favorite phantasm of the anti-biotech propaganda – is a matter of *quantity*: the alarmist fear of “uncontrolled spread” of domesticated plants or animals is unrealistic, even if operations of gene editing have slightly changed their genome, i.e. have gifted their phenotypes with desirable traits or erased undesired ones. From an empirical point of view, how “far and wide” or “uncontrollably” has this effect occurred? After many years of cultivation of “GMOs”, “examples of spontaneous transgenic crop ×WW [Wild or Weedy] hybridization remain exceedingly few […] Transgenes in WW populations are known from three cases. Evidence for hybridization is clear for those cases but not for introgression.” (Ellstrand et al. 2013. p. 340). Three cases… This non-issue was already clear many years ago: “Four different crops (oilseed rape, potato, maize and sugar beet) were grown in 12 different habitats and monitored over a period of 10 years. In no case were the genetically modified plants [herbicide tolerant and/or insect resistant] found to be more invasive or more persistent than their conventional counterparts.” (Crawley et al. 2001, Abstract).

In conclusion, there are no documented occurrences of disruption in ecological landscapes, neither from “GMOs” nor from traditional products. Due to the intrinsically limited capability of domesticated species to survive in the wild, such an awful prospect is groundless.

## The misuse of the precautionary principle

Now we come to a supposed keystone of the anti-biotech folks’ claims: “The precautionary principle is a fundamental ingredient […] in European Union (EU) legislation” (Steinbrecher and Paul, p. 40) Indeed, this link between the Precautionary principle (PP) and NBTs is baseless and the tie to the European Union is problematic.

The preliminary question is: what “EU” are we talking about? It must be explained, because there is a big gap between the detailed, science-informed guidelines on the application of the PP as issued by the European Commission and the void, merely rhetoric mentions of the PP in the “GMO” Directives.

The Commission specifies that a “decision to invoke the precautionary principle does not mean that the measures will be adopted on an arbitrary or discriminatory basis.” (European Commission 2000, Conclusion). If precautionary measures are sought on environmental or health grounds, they must always be based on “detailed scientific and other objective information” (European Commission 2000, Summary, 1). “Recourse to the precautionary principle presupposes that potentially dangerous effects deriving from a phenomenon, product or process have been identified” (European Commission 2000, Summary, 4). All these clear and straightforward indications were simply ignored in the “GMO” Directives, which show a prejudicial and unwarranted anti-biotech stance.

Furthermore, we must note a general assumption stated by the Commission, that the burden of proof is on those who call for an application of the PP: “In most cases, European consumers and the associations which represent them must demonstrate the danger associated with a procedure or a product placed on the market, except for medicines, pesticides and food additives.” (European Commission 2000, Introductory webpage). An attempt to reverse this arrow (in our case, considering that NBTs are a harmful technology, an impending *threat*^11^ for agriculture) must be rejected. That is why the subheading “The Precautionary Principle: Assessing Potential Harm Before Technologies Are Fully Developed or Deployed” (Steinbrecher and Paul, p. 45) is wrong: the trigger for the PP is not the invention of new technologies – in green biotech or in other areas – but the strong suspicion or limited evidence of danger.

Thus, critics of NBTs should become aware that, historically and theoretically, the PP in agriculture has not much to do with the vain allusions to it in the “anti-GMO” EU legislation.

Indeed, it is true that Article 1 of the basic “GMO” law, i.e. Directive 2001/18, states that “In accordance with the precautionary principle, the objective of this Directive is to […] protect human health and the environment”. (European Parliament and Council 2001). Yet, that mention of the PP is just a case of lip service from politicians to the mood of the public around the tail-end of the millennium: lawmakers “were in many countries acutely conscious in the late 1980s that the major political parties were losing ground to the Green movements; and to recapture these votes, were anxious to demonstrate their own “Green” credentials. A severely restrictive approach to the highly publicized new gene technology appeared to be a painless and popular way of doing so.” (Cantley 1995, p. 670).

The mistreatment and overstretching of the PP by the anti-biotech groups is the main reason why this good principle has become, in the view of many, a synonym for curbing agricultural progress: “[t]he so-called precautionary principle has been intelligently described as, in theory, a rational approach for balancing the risks of innovation and the risks of inactivity. In practice, it has been another heavy obstacle to the innovator.” (Cantley 2012, p. 46). Fortunately, the PP has not been misapplied everywhere: “if the current European interpretation of the PP had been in operation globally for the development of GMO technologies, we would have had no evidence for their safety or for any of the benefits.” (Tait 2016, p. 21).

In conclusion, we endorse the application of the PP according to the science-based outline provided by the European Commission, while we reject its corruption as a fig leaf by populist lawmakers.

## Nonsensical comparisons

The misreading of the PP is evident where it is stated that the downsizing of the possible danger from NBTs – importantly: NBTs as a (supposed) whole – could one day be seen as a “late lesson from early warnings”, i.e. a case where “early indications of harm were neglected with serious consequences. One of the most graphic is that of asbestos” (Steinbrecher and Paul, p. 40). The proposed parallel is bewildering. If we were discussing tragic mistakes that were made in the history of public health, we agree that quoting the case of asbestos would be appropriate. Instead, there isn’t the slightest *logical* link between one toxic mineral and a lot of different techniques which may be applied in order to create a multitude of positive traits in organisms as diverse as can be imagined, or to erase some unwanted characteristics from some of them. Asbestos is *one* thing – a dangerous substance; NBTs is a generic notion, whose sole semantic use is to indicate very different operations that, in agri-food biotech, point to very different aims.

This kind of outlandish analogy is not new. During the discussions that eventually led to the approval of the Cartagena Protocol on Biosafety, one of the proposed drafts was based on the structure of the Basel Convention on the Control of Transboundary Movements of Hazardous Wastes and Their Disposal. (Hobbs et al. 2005, p. 287–288) At any given moment, huge cargo boats are crossing the Atlantic, full of “LMO”, i.e. Living Modified Organisms – the expression used in the Biosafety Protocol to indicate seeds: it is mostly soybeans and maize grains for the livestock in Europe, where their cultivation is forbidden while importation is allowed – talk about regulatory coherence... Predictably, nobody cares: no reasonable person fears that transporting rDNA feed could be equated to moving lethal nuclear leftovers, or… asbestos.

One of the negotiators of the Protocol of Nagoya – Kuala Lumpur (a supplement to the Cartagena Protocol) is a legal expert, the editor of a book which brings together the contributions of various key players in the discussions; several years after the coming into force of the first Protocol, he wrote: “unlike oil spills polluting the ocean or nuclear power plant accidents spreading radioactive material, there has not yet been a scientifically confirmed case of environmental damage caused by LMOs. The treaty negotiators were tackling a hypothetical problem of environmental damage that may eventually be caused by LMOs without any actual experience of it.” (Shibata 2013, p. 9) Thus, a protagonist confesses the inconsistency of the subject of his work, confirming that any comparison between the real damage due to pollution from hydrocarbons or radioactive leaks and the hypothetical risks from “LMOs” (including international transport) is nonsensical.

## Epistemological inconsistencies

A further attempt is made to justify the disquieting narrative we are examining. Indeed, one may wonder why gloomy images of environmental turmoil and health hazards should be implied in agri-food novelties if they are obtained via NBTs (and rDNA), while products from older techniques – which may be very similar, as far as their actual characteristics are concerned - do not raise any concerns.

“These risks [linked to NBTs] cannot be considered equivalent to those that emerge from conventional breeding or random mutagenesis: Here the cells and the organisms have different means of regulating changes in the genome (random mutations or new gene combinations) so that the phenotype of the plants and animals is very often not changed, or only changed within certain parameters. New biological traits that emerge can adapt over longer periods of time to the environment. These natural mechanisms of genetic regulation are overridden by methods of genetic technology e.g. through simultaneous changes at several genome locations on different chromosomes, and the mass release of organisms with biological traits that have not been tested in the evolutionary process.” (Then and Bauer-Panskus 2017, p. 6).

Since this long paragraph, which is placed at the end of the Summary in the document we are considering, seems to be central to the criticism of genome engineering (“GMO” and NBTs alike) by the opponents, we must examine it carefully, distinguish several points which are improperly mixed up:

* We do not see how conventional breeding methods “have different means of regulating changes in the genome (random mutations or new gene combinations)”: the fact is that traditional crossing/hybridization and physical/chemical mutagenesis are blind processes, in which the genomes are blended (in sexual crossing) or scrambled (in mutagenesis) and, if fortune favors, naturally reorganize with the possible emergence of new traits that breeders can appreciate and select. The very same biological mechanisms kick off in (much more precise) experiments with rDNA or NBTs, without triggering any different or special genotypic-phenotypic “regulation” that would make them diverse from traditional biotechnologies.

* In all these operations, the phenotypes of the plants and animals are *always* changed, of course: if no changes occurred, there would be no breeding, i.e. no new interesting organisms would be generated. It should also be evident that this happens “within certain parameters”: cucumber seeds which are bombarded with radiation will never produce apricot plants. It is not explained how unpredictable changes, which are caused by the application of older methods, should distinctly differ from those generated by newer techniques; if anything, as we have already explained, NBTs generate changes inside narrower, not wider, parameters than traditional techniques.

* Thus, the “simultaneous changes at several genome locations on different chromosomes” that are allegedly typical of “genetic technology” may be evident in new cultivars from traditional breeding methods, which imply massive rearrangements of genomes. And, in any case, this is not a matter of concern, insofar as tests and exams – which, remember, are normally not required for enhanced organisms that are not “GMO” – show no undesired effects *in the phenotype*. Again, the antis fail to acknowledge that what matters is the product, not the process.

* As for the “longer periods of time” that cultivars obtained via traditional methods declaredly enjoy for their environmental adaptation, a quick look at the lists of registered plants, e.g. in the Mutant Variety Database (FAO-IAEA 2018), shows that new organisms are continuously added, while older items are gradually abandoned – breeders worldwide are very active in their job: therefore, the supposed long-term adaptation to the environment of varieties obtained via traditional methods is not real – and it will not be the case for outcomes from the NBTs. Critics are simply not aware that the mixed salad they have tasted today may have been composed with vegetables whose “prototypes” were created in the last few months – literally.

* The idea that enhanced cultivars or animals obtained via traditional methods are “tested by evolutionary process” shows a perfect incomprehension of what agriculture is: evolution happens in the wild, over millennia, ages, epochs; the few natural mutations that may occur in greenhouses or fields, over years or decades, are normally irrelevant for cultivation/farming operations and results. In Darwinian terms, what is at work in breeding new plants, animals or microorganisms is not natural selection, but artificial selection: the aim of breeding efforts is to twist nature in order to modify its products according to human interests. In other words, agriculture is the realization of a highly unnatural redirection of natural, spontaneous paths toward results that humans want. Therefore, the reference to the evolutionary process is inappropriate.

* Finally, note that no reference is made to scientific texts in support of the confused statements that we have dissected.

Some better explanation could be hoped for from a chapter of the same document, which deals a bit longer with the “comparison” of NBTs “with random mutagenesis” (the following quotations are from Then and Bauer-Panskus 2017, p. 17–19): but the text is disconcerting. After having correctly pointed out that “many spontaneous or induced changes in the genome (mutations, ‘jumping genes’, changed gene activity) are not really random but are subject to regulation mechanisms in the cells/organisms”, it is claimed that “[m]ethods of genetic engineering try to bypass these mechanisms to achieve the desired result.” Yet, the existence of inner capacity of genomes to reorganize after major genetic changes and mixtures, like those generated by sexual crossing, is exactly the reason why traditional physical/chemical mutagenesis, whose operations stir the DNA at random, sometimes generate specimens that not only survive the shuffling, but show useful new traits – the blessed breeder hit the jackpot. In what sense NBTs, with their augmented precision, are supposed to “bypass these mechanisms” and why this alleged stratagem should be a matter of concern, remains unclear.

In sum, the demand for special risk assessment for certain newer agri-food biotech applications has no rational rationale.

To be clear, we are not rejecting the request for “comprehensive risk assessment” of products obtained via NBTs: on the contrary, we believe that an adequate evaluation of *any* agricultural novelty should be made before marketing, irrespectively of the process(es) used by breeders. What we don’t understand are the alleged scientific reasons why, in the problematic document we are examining, it is maintained that tests and exams should be mandated for outcomes of genome editing – in particular when multiple and simultaneous precise changes are achieved – as if peculiar, higher risks were involved.

Ironically, we agree with the authors when they declare that “[f]rom a scientific point of view, it is not understandable why it matters how extensive the changed section is and whether DNA is inserted, changed or removed. All such changes have to undergo a detailed risk assessment.” Making a simple deduction from their own statement, they would not ask for sectarian over-regulation for products from rDNA and NBTs; rather they would call for rebalancing the under-regulation of products from conventional breeding techniques (comprising traditional mutagenesis). Curiously, and certainly inadvertently, these authors declare that “GMO” means nothing, as far as a science-informed regulation is concerned.

All in all, the attempt by opponents of NBTs, as it was for “GMO”, to argue for theoretical grounds that demonstrate their inevitable dangerous nature ends up in contradictions or non-sequiturs: when scientific papers are quoted, their meaning is often misunderstood; and certain statements that may appear science-sounding do not even survive a first step of scrutiny.

## Conclusion

We must reaffirm that our criticism is logically placed *before* any debate regarding the best utilization of (products from) the NBTs.

Let’s imagine that NBT opponents correctly explain the basic science of those breeding methods: they would have every right to be against them (i.e. anti-biotechnology), for ethical, metaphysical, political or other reasons. But this is not so: their description of NBTs is mistaken in several ways, and this misapprehension pollutes any subsequent reasoning.

One may wonder why anti-biotech groups present such fallacious arguments. What are the possible motivations for certain groups to embrace views which are utterly anti-scientific? What mental mechanisms are operating? In our opinion, it is not (mostly) a matter of wrong-headed reasoning, but biases are passed off as scientifically grounded arguments in the pursuit of pushing a political agenda: science has a great appeal to the public and to decision-makers, but it is difficult for non-specialists, even for scholars who are not knowledgeable in biology and genetics, to separate the wheat from the chaff. Cherry-picking half-truths or developing scientific-sounding pseudo-arguments and peddling them as sound evidence – or as indications of “risk” – is a winning strategy, if the decades-long story of the relentless opposition to so-called “GMOs” can teach us anything.

Even more unfortunately, there are other examples of such biases at work: the resurgence of the anti-vaccine movement is nourished by fake news, but it originated in a scientific paper which advanced incorrect suppositions regarding possible negative effects of certain preventive treatments. As it happened, the self-correcting mechanisms which protect the scientific community led to an almost immediate rejection of the mistaken positions: but the fact the paper was retracted only fuelled the conspiracy theory of groups that are very good at exploiting pseudo-science in their propaganda.

Going back to NBTs: witnessing the misplaced but effective attacks on their work, many life scientists and breeders still hope that NBTs will not suffer the regulatory destiny of “GMOs”; but the panorama is complex and ever shifting. While several countries seem unwilling to impose the “GMO” straitjacket on the newest methods, the recent ruling by the Court of Justice of the European Union (CJEU) on mutagenesis using gene edition tools is discouraging – at least for the Old Continent: contrary to the overwhelming majority of the scientific community and to the opinion of the Advocate General, the CJEU issued a binding sentence (Court of Justice 2018) that equates the products deriving from mutagenetic NBTs with “GMOs”: falling under the umbrella of the infamous Directive 2001/18, new cultivars obtained via such applications are destined to enter the “GMO” regulatory quagmire. The state of things is clear: judges can issue decisions that are widely judged as unscientific (see e.g. Urnov et al. 2018 and www.sciencemediacentre.org/expert-reaction-to-court-of-justice-of-the-european-union-ruling-that-gmo-rules-should-cover-plant-genome-editing-techniques. Accessed 20 September 2018). We have no evidence as to whether that ruling was influenced by incorrect presentation of scientific facts – but such a suspicion cannot be excluded.

## Footnotes

1 We will insist on using this acronym in inverted commas, to constantly underline its shakiness. Interestingly, the specialists of the European Scientific Advice Mechanism refer to rDNA as “Established Techniques of Genetic Modification (ETGM)” (European Commission, Scientific Advice Mechanism 2017): such definition sounds correct and may be useful in order to avoid the “GMO” semantic trap, i.e. the negative halo which has been constructed around these products by the successful anti-biotech propaganda.

2 Sometimes, limiting the perimeter, we could talk about “New Plant Breeding Techniques”, “NPBTs”. Another expression and acronym to indicate NBTs has also been suggested, i.e. “New genetic modification techniques (NGMTs)”. (ENSSER 2017)

3 Since this paper is mostly directed at scholars in social sciences, yet fairly knowledgeable in genetics and biology, the references we provide do not list all the technical literature; where available, we indicate articles which are readable by non-specialists, while being scientifically sound.

4 Even a single “letter” can now be replaced, without cutting the DNA strands: it is called “base editing”. (Dolgin 2017, Cohen 2017)

5 “Chromatic” partitions may comprise from four (www.pharmtech.com/four-colours-biotechnologyy) to ten (www.ejbiotechnology.info/index.php/ejbiotechnology/article/view/1114/1496) colors.

6 Such consilience has been questioned (Krimsky 2015), but from a wrong perspective (see Tagliabue 2016).

7 Here is one of the many examples: “the Xa21 disease resistance gene in rice has been bred into commercial rice cultivars using both rDNA and by traditional crossing. The traditional line can proceed to market with no regulatory scrutiny, while the essentially identical line produced using rDNA languishes.” (McHughen 2016, p. 137)

8 The insistence on the possible negative fall-outs from the smallest mutations overshadows the opposite perspective, i.e. that a SNP (“single nucleotide polymorphism”) can give birth to very positive outcomes: this is what happened e.g. during the domestication of rice, when farmers selected rare plants that, due to a lucky accident, were unable to drop their seeds; therefore, multiplied in the paddies, that kind of cultivar made it easier to obtain a more abundant harvest. Such a tiny mutation, that would have impaired the reproduction in the wild of the plants bearing that trait (Konishi et al. 2006) was welcomed by breeders. Of course, the fact that the mutation happened by chance in nature has no relevance for its positive effect on agriculture: similar mutations leading to useful traits, if induced by a NBT - or any other human-driven method, for that matter – must be appreciated.

9 The possible problem was actually foreseen by experimenters; it was then questioned by another study (Lee et al. 2013). In any case, the product was discarded.

10 To be precise, the pea case led to some temporary reactions: a Greenpeace campaigner declared that “Withdrawing a failure doesn’t show the success of the regulatory system.” (www.agbioworld.org/newsletter_wm/index.php?caseid=archive&newsid=2445. Accessed 20 May 2018). One may wonder what it takes to satisfy such an irredeemable disgruntlement. After that feeble protest, the episode was forgotten.

11 “Threat” is the word used in the original definition of the PP: see the Rio Declaration on Environment and Development (United Nations 1992): only a serious danger – although not (yet) scientifically certain - can be the trigger for the adoption of precautionary measures.
